# Unusually Divergent Ubiquitin Genes and Proteins in *Plasmodium* Species

**DOI:** 10.1093/gbe/evad137

**Published:** 2023-07-22

**Authors:** Thomas Dalhuisen, Lindsey J Plenderleith, Ismail Ursani, Nisha Philip, Beatrice H Hahn, Paul M Sharp

**Affiliations:** Institute of Ecology and Evolution, University of Edinburgh, Edinburgh, United Kingdom; Centre for Immunity, Infection and Evolution, University of Edinburgh, Edinburgh, United Kingdom; Institute of Ecology and Evolution, University of Edinburgh, Edinburgh, United Kingdom; Centre for Immunity, Infection and Evolution, University of Edinburgh, Edinburgh, United Kingdom; Institute of Ecology and Evolution, University of Edinburgh, Edinburgh, United Kingdom; Centre for Immunity, Infection and Evolution, University of Edinburgh, Edinburgh, United Kingdom; Institute of Immunology and Infection Research, University of Edinburgh, Edinburgh, United Kingdom; Departments of Medicine and Microbiology, University of Pennsylvania, Philadelphia, Pennsylvania, USA; Institute of Ecology and Evolution, University of Edinburgh, Edinburgh, United Kingdom; Centre for Immunity, Infection and Evolution, University of Edinburgh, Edinburgh, United Kingdom

**Keywords:** ubiquitin, concerted evolution, *Plasmodium ovale*, *Plasmodium vivax*

## Abstract

Ubiquitin is an extraordinarily highly conserved 76 amino acid protein encoded by three different types of gene, where the primary translation products are fusions either of ubiquitin with one of two ribosomal proteins (RPs) or of multiple ubiquitin monomers from head to tail. Here, we investigate the evolution of ubiquitin genes in mammalian malaria parasites (*Plasmodium* species). The ubiquitin encoded by the RPS27a fusion gene is highly divergent, as previously found in a variety of protists. However, we also find that two other forms of divergent ubiquitin sequence, each previously thought to be extremely rare, have arisen recently during the divergence of *Plasmodium* subgenera. On two occasions, in two distinct lineages, the ubiquitin encoded by the RPL40 fusion gene has rapidly diverged. In addition, in one of these lineages, the polyubiquitin genes have undergone a single codon insertion, previously considered a unique feature of Rhizaria. There has been disagreement whether the multiple ubiquitin coding repeats within a genome exhibit concerted evolution or undergo a birth-and-death process; the *Plasmodium* ubiquitin genes show clear signs of concerted evolution, including the spread of this codon insertion to multiple repeats within the polyubiquitin gene.

**Significance**

Ubiquitin is a multifunctional protein, encoded as three different fusion products. Ubiquitin is generally extraordinarily highly conserved, but here, we find that the proteins encoded by some malaria parasites (*Plasmodium* species) have undergone extremely unusual changes. This raises questions about the function(s) of these divergent forms of ubiquitin and the factors allowing the escape from sequence conservation. The multiple ubiquitin coding sequences in these species have undergone concerted evolution.

## Introduction

**Fig. 1.**
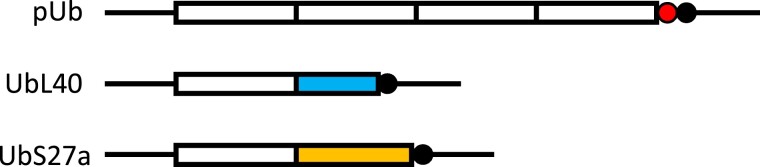
Schematic of the organization of gene sequences encoding ubiquitin in *Plasmodium*. White blocks denote the 76 codons for ubiquitin. In UbL40, the second (blue) block is the 52 codons for RP L40; in UbS27a, the second (yellow) block is the 77 codons for RP S27*a*. Black circles indicate the translation termination codon. In the polyubiquitin (pUb) genes, the number of repeats of the ubiquitin coding unit (shown as four here) varies from one to eight, and there is a single extra codon (red circle) after the last unit.

Ubiquitin is a remarkable protein, encoded by very unusual genes. This small (76 amino acid) protein is a post-translational modifier of proteins, first identified for its essential role in proteasome-dependent protein degradation, but subsequently shown to be also involved in numerous other cellular processes, including protein trafficking, chromatin dynamics, cell cycle regulation, and DNA repair (Kwon and Ciechanover 2017; Dougherty et al. 2020). Ubiquitin is one of the most conserved proteins known, rivaling histone 4 in this regard (Sharp and Li 1987a). Initial surveys found that most eukaryotes have three kinds of ubiquitin genes: 1) a polyubiquitin gene comprised of multiple repeats of the 76 codons for ubiquitin, followed by a single extra codon before the translation termination codon, and genes encoding a single ubiquitin fused to either 2) a 52 amino acid C-terminal extension (CEP52) or 3) an 80 amino acid C-terminal extension (CEP80) (Ozkaynak et al. 1987). CEP52 and CEP80 were subsequently identified as being ribosomal proteins (RPs) L40 (Chan et al. 1995) and S27a (Redman and Rechsteiner 1989), respectively. (Here, we will refer to these three kinds of genes as pUb, UbL40, and UbS27a, respectively; see fig. 1.) In some species, there is more than one copy of one or more of these three kinds of genes.

Apart from extreme protein sequence conservation, another interesting evolutionary feature of ubiquitin is that the repeated copies of the coding sequence appear to exhibit “concerted evolution,” such that paralogous copies within a species are more similar than orthologous copies in other species (Zimmer et al. 1980; Elder and Turner 1995). For example, in comparisons of human, yeast (*Saccharomyces cerevisiae*), and a slime mold (*Dictyostelium discoideum*), there were small differences in ubiquitin sequences among species, but within each species, the ubiquitin proteins encoded by the three different kinds of genes were identical (Sharp and Li 1987b). More strikingly, synonymous nucleotide substitutions accumulate within the polyubiquitin gene sequences, but the different repeats within a species are more similar than repeats compared between species (Sharp and Li 1987a). The repeats within the polyubiquitin gene appear to undergo unequal crossing-over, as polymorphic variants differing in the number of repeat copies are known (Dworkin-Rastl et al. 1984), but any recombination between the different ubiquitin loci must involve gene conversion. Concerted evolution of ubiquitin genes had been reported in a number of diverse species, including animals, fungi, plants, and some protists (Sharp and Li 1987b; Tan et al. 1993; Keeling and Doolittle 1995; Zhou and Regan 1995; Nenoi et al. 1998). However, it was subsequently argued that the semblance of concerted evolution was simply due to a strong purifying selection on the ubiquitin protein sequence, that the number of synonymous nucleotide differences between ubiquitin coding repeats is inconsistent with concerted evolution, and that the ubiquitin gene family is subject to “birth-and-death evolution” where repeats share similarity due to recent duplication (Nei et al. 2000; Nei and Rooney 2005). A third perspective suggested that the polyubiquitin locus is a selfish gene, acting as the template for recombination events that result in the conservation of the protein encoded by the different ubiquitin loci (Catic and Ploegh 2005). It had been found that the UbS27a gene of *Caenorhabditis* nematodes encodes a highly divergent ubiquitin sequence (Jones and Candido 1993), and a similar observation was made for UbS27a genes from 12 other species, including 9 species from 6 different phyla of protists (Catic and Ploegh 2005). This was interpreted as evidence that failure to recombine had allowed the divergence of the UbS27a ubiquitin sequence (Catic and Ploegh 2005).

*Plasmodium* species are members of the protist phylum Apicomplexa. Six *Plasmodium* species commonly cause malaria in humans, and so there has been much interest in genetic analyses of these parasites and their relatives infecting other mammals. The polyubiquitin gene of one of the human parasites, *Plasmodium falciparum*, was found to contain five repeats, encoding a protein with only one amino acid different from that of vertebrates (Horrocks and Newbold 2000). The two ubiquitin-RP fusion genes were later identified (Catic and Ploegh 2005) from the *P. falciparum* 3D7 genome sequence (Gardner et al. 2002); *P. falciparum* was among those species in which the ubiquitin encoded by the UbS27a gene was highly divergent (Catic and Ploegh 2005). However, there appears to have been no other studies of ubiquitin genes, or their evolution, in *Plasmodium*. In recent years, genome sequences of numerous species of mammalian *Plasmodium* species have been determined. Here, we mine these data to examine the evolution of the ubiquitin gene family in *Plasmodium*. These sequences provide clear evidence of concerted evolution and undermine the birth-and-death interpretation. Remarkably, they also reveal that two aberrant forms of ubiquitin, previously thought to be extremely rare, have each coincidentally arisen very recently during the diversification of the mammalian *Plasmodium* species.

### Ubiquitin Genes in Mammalian *Plasmodium*

**Fig. 2.**
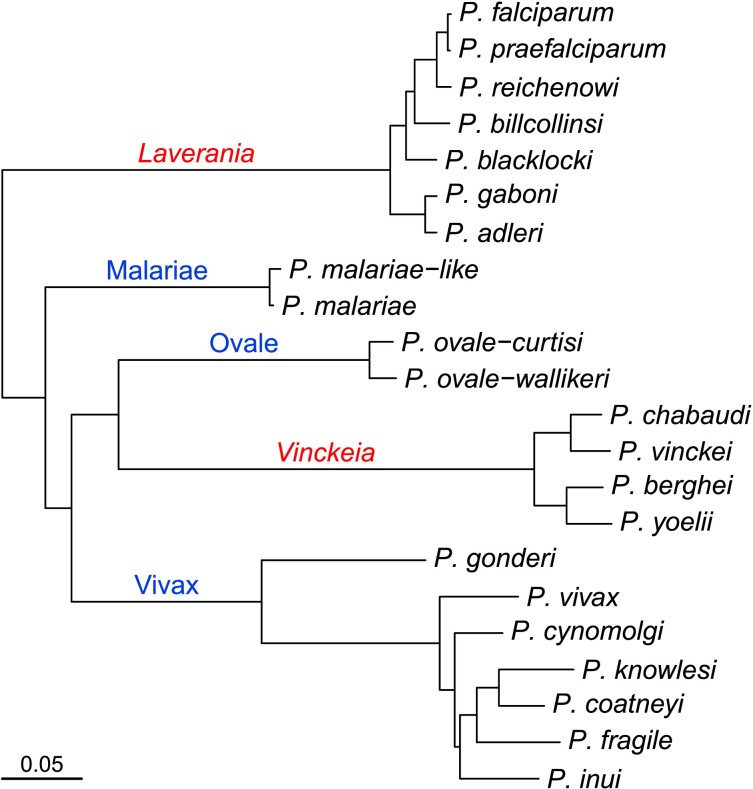
Phylogenetic relationships among mammalian *Plasmodium* species. Names above branches indicate the five major lineages referred to in this study; names in italics (in red) are designated subgenera, other names (in blue) are coined here for convenience. The phylogeny was derived from maximum likelihood analysis of 2,773 concatenated proteins (a total of 1,095,696 amino acid sites) encoded by the nuclear genome, and rooted using *P. relictum* (not shown) as an outgroup.

We investigated the ubiquitin genes in 22 species of mammalian *Plasmodium* species for which complete genome sequences are available (fig. 2). The mammalian parasites form a clade separate from *Plasmodium* species infecting birds and reptiles, and we used sequences from a bird parasite, *Plasmodium relictum*, as an outgroup. These mammalian parasites fall into five distinct lineages (Sharp et al. 2020). Two of these have previously been designated as subgenera (Garnham 1964): *Laverania*, comprised of species infecting African apes, from one of which the human parasite *P. falciparum* was recently derived (Liu et al. 2010), and *Vinckeia*, comprised (mainly) of rodent parasites. The other three lineages are all currently placed in the subgenus *Plasmodium*, although recent phylogenetic analyses have shown that they are not monophyletic (Rutledge et al. 2017; Sharp et al. 2020); here, we refer to these as the Malariae, Ovale, and Vivax lineages (fig. 2). The Malariae lineage includes the human parasite *Plasmodium malariae*, as well as closely related species found in African apes (Rutledge et al. 2017; Plenderleith et al. 2022). The Ovale lineage comprises two cryptic, sympatric, but clearly genetically distinct species (Sutherland et al. 2010) that infect humans and also African apes (Sharp et al. 2020); these are *Plasmodium ovale-curtisi* and *P. ovale-wallikeri* (note that we have inserted hyphens in these names to emphasize that these are not subspecies). Here, the Vivax lineage comprises a parasite from African monkeys (*Plasmodium gonderi*), a parasite that infects humans and African apes (*Plasmodium vivax*), and a clade of parasites from Southeast Asia that infect macaques; one of those parasites (*Plasmodium knowlesi*) has become recognized as a very frequent zoonotic infection of humans (Cox-Singh et al. 2008).

All of the *Plasmodium* genomes analyzed here contain only one copy of each type of ubiquitin gene, and we detected no pseudogenes. Gaps in genome assemblies mean that the pUb gene in *Plasmodium fragile* and the UbS27a gene in *P. malariae*-like are incomplete. The polyubiquitin genes typically contain four or five repeats, although the number ranges from one (in some strains of *Plasmodium berghei*) to eight (in *P. knowlesi*) (supplementary table S1, Supplementary Material online). All of the pUb genes have a single intron, interrupting codon 10 of the first repeat. The UbL40 genes have a single intron, after codon 69 of the ubiquitin coding sequence, while the UbS27a genes have two introns, interrupting codon 35 and after codon 63 of the ubiquitin coding sequence.

These 22 *Plasmodium* species all have 14 chromosomes, with extensive blocks of synteny evident among the major lineages (Carlton et al. 2008). While the three ubiquitin genes are, in some cases, assigned to different chromosomes in different lineages, they each lie within a region of synteny extending across at least 100 genes, found in all five lineages as well as the outgroup *P. relictum* (supplementary table S2, Supplementary Material online). Thus, there is no evidence of duplication or loss (birth or death) of ubiquitin genes during the diversification of these species.

### Ubiquitin Protein Evolution in *Plasmodium*

**Fig. 3.**
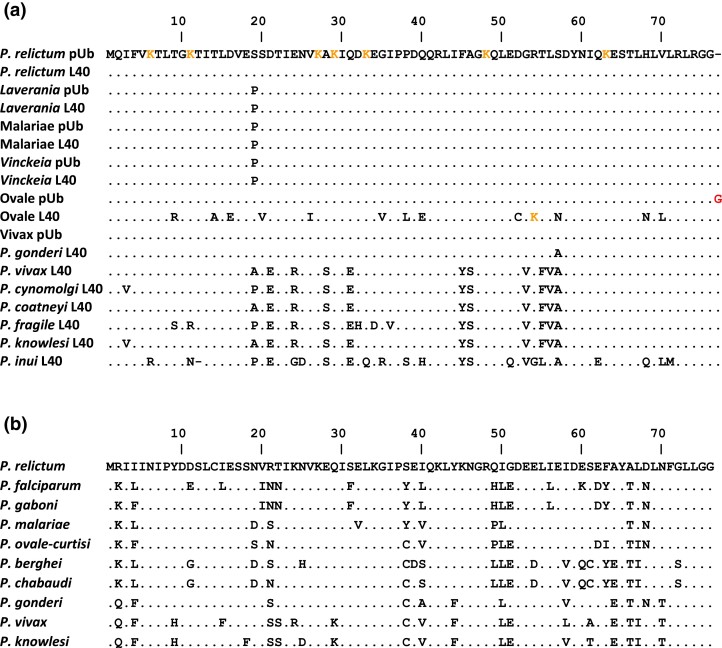
Alignment of *Plasmodium* ubiquitin sequences. (*a*) Sequences encoded by polyubiquitin (pUb) and UbL40 genes; only differences from the *P. relictum* (outgroup) sequence are shown, and rare variants encoded by only one repeat within the pUb gene are not shown (see supplementary fig. S1, Supplementary material online). Within each of the five lineages (*Laverania*, Malariae, *Vinckeia*, Ovale, Vivax) ubiquitin sequences encoded by the polyubiquitin genes of all species are identical. Ubiquitin sequences encoded by the UbL40 genes vary among species within the Vivax lineage but are identical among species within each of the four other lineages. Seven lysine residues involved in forming covalent linkages are shown in orange in the *P. relictum* sequence, as is an additional lysine (site 57) in Ovale UbL40; an additional terminal glycine (site 77) in Ovale pUb is shown in red. (*b*) Sequences encoded by the UbS27*a* genes; only differences from the *P. relictum* (outgroup) sequence are shown. Representative sequences from the *Laverania* (*P. falciparum* and *P. gaboni*), Malariae (*P. malariae*), Ovale (*P. ovale-curtisi*), *Vinckeia* (*P. berghei* and *P. chabaudi*), and Vivax lineages (*P. gonderi*, *P. vivax*, and *P. knowlesi*) are shown.

The ubiquitin sequences encoded by pUb genes show one fixed difference among the *Plasmodium* species: residue 19 is Pro in *Laverania*, *Vinckeia,* and the Malariae lineage, but Ser in the Vivax and Ovale lineages (fig. 3*a*). This site is also Ser in the outgroup *P. relictum*, and given their phylogeny (fig. 2), this site must have undergone multiple switches during the diversification of the mammalian parasites, followed by spread to all repeats by recombination events. There are two variants, each found in only one repeat: Ser (in place of Ala) at site 28 in the first repeat of the *P. vivax* gene (in all four strains analyzed here), and Leu (in place of Ser) at site 57 in the first repeat of both species in the Ovale lineage. The C-terminal residue encoded by the pUb genes also varies: it is Cys in *Vinckeia* but Phe in the other four lineages (and the outgroup). Otherwise, the ubiquitin sequences encoded by the pUb genes are completely conserved. However, a striking observation is that the first four (of five) ubiquitin coding repeats within the pUb gene of both *P. ovale-curtisi* and *P. ovale-wallikeri* each contains 77 codons, due to the insertion of a codon near the 3′ end; the repeats end with three Gly codons rather than two. This insertion presumably occurred in a single repeat, after the Ovale lineage split from the ancestor of the *Vinckeia* subgenus (fig. 2), and has spread to all but the fifth repeat through unequal crossing-over and/or gene conversion.

In three lineages, *Laverania*, *Vinckeia*, and Malariae, the UbL40 fusion protein sequences are completely conserved, and the ubiquitin sequences are identical to those encoded by the pUb genes. However, in the Ovale and Vivax lineages, while the RP L40 sequences are identical to those of the other three lineages, the ubiquitin sequences have diverged (fig. 3*a*). In the Ovale lineage, there are 13 differences (17.1% of residues) from the ubiquitin encoded by the pUb gene, and in the Vivax lineage (excluding *P. gonderi*), there are 11–23 differences (14.5–30.3%), but these differences are not shared between the two lineages (fig. 3*a*). These two lineages are not sister groups in the phylogeny (fig. 2) and so the release from conservation reflects two independent events. Notably, the first diverging species within the Vivax lineage, *P. gonderi*, has only one of the amino acid changes, but nine of the other changes are found in at least five of the other six species, and the *P. vivax* and *Plasmodium coatneyi* sequences are identical (fig. 3*a*); thus, most of this divergence occurred in a burst on the branch after the split of the ancestor of *P. gonderi* and before the divergence of *P. vivax*.

**Fig. 4.**
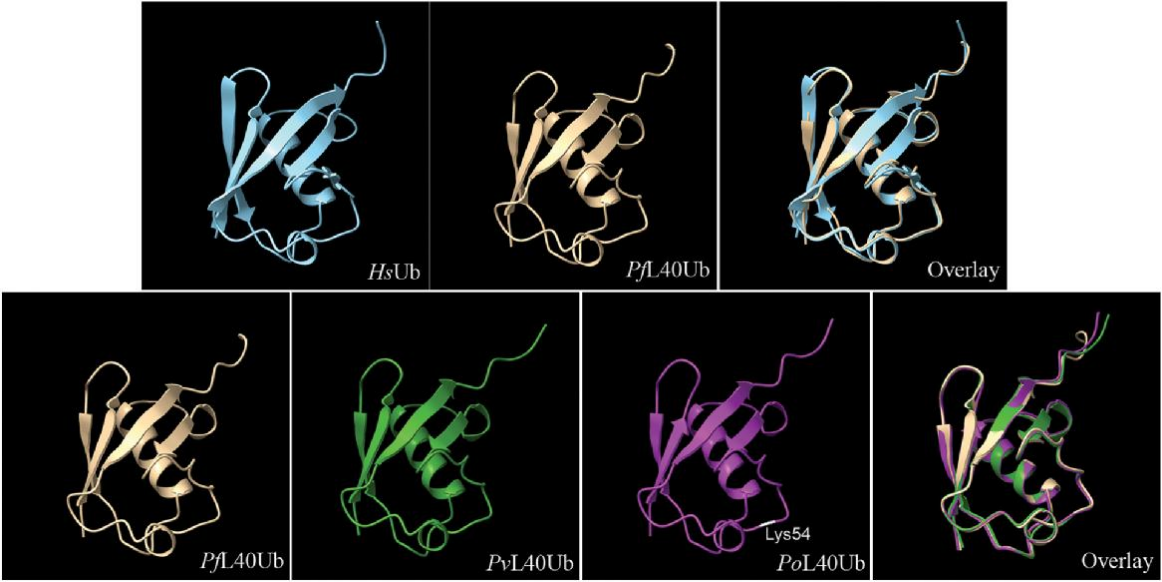
Comparison of predicted *Plasmodium* ubiquitin structures to the crystal structure of human ubiquitin (*Hs*Ub). The *Plasmodium* ubiquitins are those encoded by the pUb and UbL40 genes of *P. falciparum* (*Pf*L40Ub; one amino acid difference from human ubiquitin), *P. vivax* (*Pv*L40Ub; 12 differences from human), and *P. ovale-wallikeri* (*Po*L40Ub; 13 differences from human, with Lys54 highlighted). The overlay panels at the right show the extreme similarity between the human and *P. falciparum* structures (upper row), and among the three *Plasmodium* structures (lower row).

We compared the predicted structures of the ubiquitin encoded by the *Plasmodium* pUb genes (represented by *P. falciparum*) to the known structure of human ubiquitin, and the predicted structures of the divergent ubiquitins encoded by the UbL40 genes of the Vivax and Ovale lineages (represented by *P. vivax* and *P. ovale-wallikeri*, respectively) to that of the *P. falciparum* pUb gene (fig. 4). As expected, with only one amino acid difference, the *P. falciparum* structure is predicted to be almost identical to that of human ubiquitin. More surprisingly, an overlay of the divergent UbL40-encoded ubiquitins from *P. vivax* and *P. ovale-wallikeri* with the conserved ubiquitin from *P. falciparum* also reveals near identical structures.

The ubiquitin encoded by the UbS27a genes of all of the mammalian *Plasmodium* species is conserved in length, but extremely divergent in sequence; for example, the *P. falciparum* sequence shares only 24 of 76 residues with the ubiquitin encoded by the *Laverania* pUb genes. Sequences from the various mammalian *Plasmodium* species (fig. 3*b*) differ by as many as 28 residues (36.8%). In contrast, the RP S27a sequences encoded by the UbS27a genes are more conserved, differing by at most seven residues (9.6%).

### Ubiquitin Gene Evolution in *Plasmodium*

**Fig. 5.**
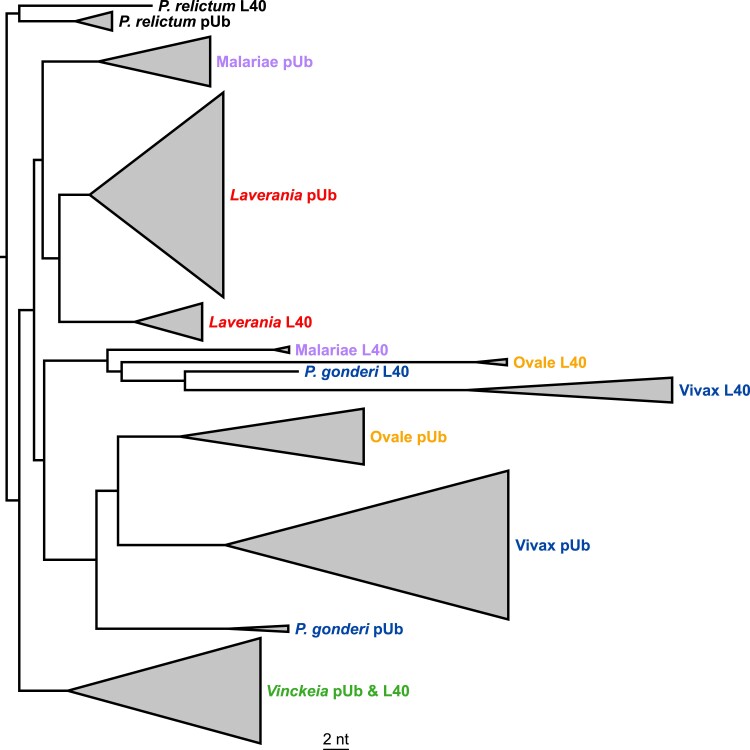
Overview of levels of similarity among the DNA sequences of ubiquitin coding units of pUb and UbL40 genes from 22 mammalian *Plasmodium* species. Triangles summarize the diversity of the pUb or UbL40 (L40) units within clades. *Plasmodium relictum* was used as an outgroup. The scale bar indicates two nucleotide substitutions per site.

**Fig. 6.**
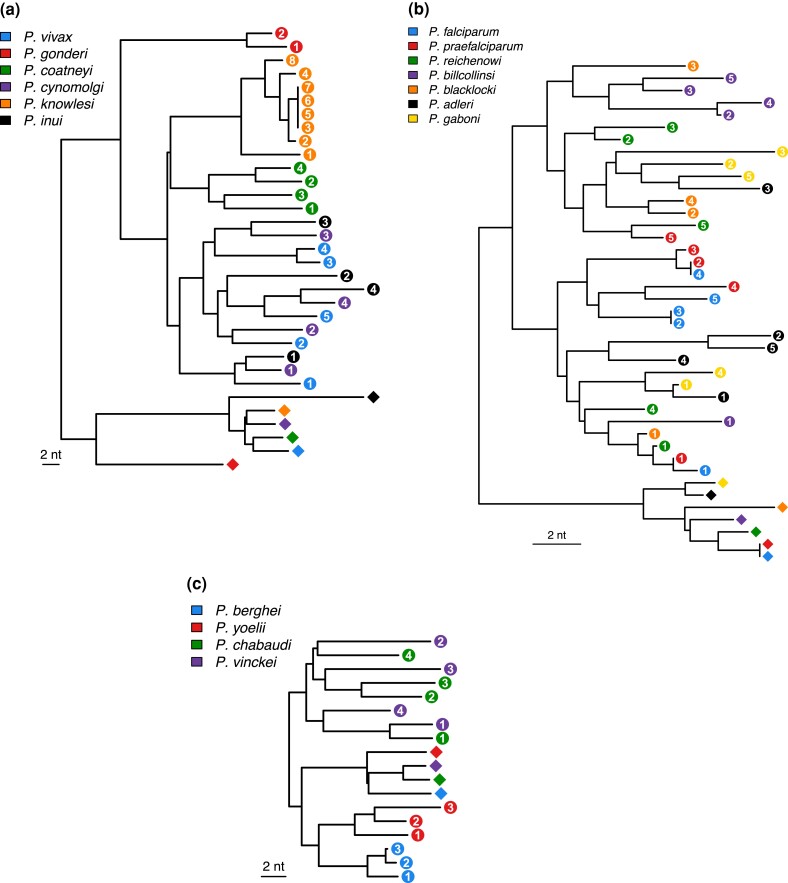
Dendrograms summarizing levels of similarity among the DNA sequences of ubiquitin coding units from pUb (circles, numbered according to position within the locus) and UbL40 genes (diamonds). Units are color-coded according to species. (*a*) Six species from the Vivax lineage. (*b*) Seven species from the *Laverania* subgenus. (*c*) Four species from the *Vinckeia* subgenus. The scale bar indicates two nucleotide substitutions per site.

While the ubiquitin sequences encoded by the pUb and L40 genes are generally highly conserved (fig. 3*a*), the nucleotide sequences of these genes exhibit substantial divergence due to (presumably neutral) synonymous substitutions. These sequences provide extensive evidence of concerted evolution among the repeats within the pUb loci. The number of nucleotide differences between repeats from the same pUb locus is generally much lower than the number of differences between repeats compared between species, especially when the species are more distantly related (i.e., belonging to different major lineages). For example, the five repeats from the *P. falciparum* pUb locus differ on average at 7.4 sites but differ at a minimum of 21 sites from repeats in *P. malariae*, and at least 33, 41, and 23 sites from those in *P. ovale-wallikeri*, *P. vivax*, and *P. berghei*, respectively. Similarly, the five repeats from the *P. malariae* pUb locus differ on average at 9.4 sites but differ at a minimum of 27, 41, and 24 sites from the pUb repeats of *P. ovale-wallikeri*, *P. vivax,* and *P. berghei*, respectively. (Details of the nucleotide differences, within and between loci and species, are given in supplementary table S3, Supplementary Material online.) This can be captured in a dendrogram summarizing the levels of similarities among the ubiquitin coding sequences from the pUB and L40 loci; the repeats from the five major lineages each form separate clusters (fig. 5), indicating that there has been within-species recombination involving these repeats subsequent to the divergence of the ancestors of these lineages. In the case of the *Vinckeia* subgenus, the ubiquitin coding sequences from the UbL40 locus also fall within the cluster of sequences from the pUb loci (fig. 5; see also fig. 6c), suggesting that there has been recent exchange between the two different loci.

It is notable that the branch lengths within the Vivax lineage are longer than those in other lineages (fig. 5). This is associated with a shift to higher G + C content in the genomes of the Vivax lineage, particularly after the divergence from *P. gonderi* (supplementary table S4, Supplementary Material online). For example, while GC3s values (the G + C content at synonymously variable third positions of codons) are around 0.18–0.19 for the pUb and UbL40 genes in Laverania, they are around 0.50 in *P. vivax*. However, parallel A/T to G/C mutations in different ubiquitin repeats would be insufficient to explain the appearance of concerted evolution. Furthermore, the pUb genes in the *Laverania* and Malariae lineages have very similar GC3s values but form separate clusters (fig. 5).

Detailed inspection of the levels of similarity among repeats within lineages reveals numerous instances of concerted evolution. For example, within the Vivax lineage, all eight repeats in *P. knowlesi*, and all four repeats in *P. coatneyi*, are more similar to each other than to any repeats from the pUb loci of other closely related species (fig. 6*a*). Within the *Laverania* there are no examples as extreme as this, but nevertheless clear indications of exchange, for example, among four of the five repeats in the *Plasmodium billcollinsi* gene cluster (fig. 6*b*). Exceptions to this typically involve the first repeats of the pUb loci; for example, in the *Laverania* the first repeats from five species form a cluster (fig. 6*b*). Another sign of exchange comes from comparison of the extremely closely related species *P. falciparum* and *P. praefalciparum*: both have five repeats in the pUb gene, but the fourth repeat in *P. falciparum* is identical to the second repeat in *P. praefalciparum*. The dendrograms are only indicative of the levels of similarity among repeats and cannot convey the full evolutionary history of the repeats, because the recombination events would not have boundaries coinciding with the ends of the repeats. There are clear signs of recent homogenization of the ubiquitin coding repeats, where the boundaries appear to be in the middle of the repeats. For example, in the five repeats within the *P. falciparum* 3D7 pUb gene, the first repeat differs from the consensus at five sites in the first 72 (of 228) nucleotides, but there are no further differences from the consensus until site 87 of the fourth repeat (supplementary fig. S2, Supplementary Material online). Furthermore, among the 16 sites which vary among the 5 repeats, 11 are variants present in a single repeat, while 5 are present in 2 (the fourth and fifth) repeats; these 5 sites are in a block, providing clear evidence of exchange among the repeats (supplementary fig. S2, Supplementary Material online). Similar examples exist in the pUb genes of the other *Plasmodium* species.

## Discussion

We have characterized the ubiquitin gene family in mammalian *Plasmodium* species, examining the evolution of these genes, and the proteins they encode. With the exception of rare variants, the ubiquitin proteins encoded by the polyubiquitin (pUb) genes vary by only one amino acid across this clade. Furthermore, these *Plasmodium* ubiquitin sequences differ from that of humans by only one or two residues; this reflects extraordinary conservation, since Alveolates (including Apicomplexa) and Opisthokonts (including metazoa) may have been diverging since the last common ancestor of all eukaryotes (Keeling and Burki 2019). By contrast, the archetypical highly conserved protein, histone H4 (Dickerson 1971), differs at eight sites (7.8%) between *Plasmodium* and humans. Extreme conservation is typically interpreted as evidence that the precise protein sequence is critical for function. Paradoxically, in yeast, it was found that each of 47 of 63 residues on the surface of ubiquitin could be replaced without disrupting vegetative growth (Sloper-Mould et al. 2001); nevertheless, it is quite possible that growth under laboratory conditions fails to detect subtle fitness differences that would prevent the spread of mutations in the wild.

*Plasmodium falciparum* was among those species in which the ubiquitin encoded by the UbS27a gene was previously found to be highly divergent (Catic and Ploegh 2005). Since this was also true of the two other members of the Apicomplexa included in that analysis (*Cryptosporidium hominis* and *Toxoplasma gondii*), it is perhaps unsurprising to find that the UbS27a-encoded “ubiquitin” is highly divergent across all of the *Plasmodium* species here. However, two other forms of variant ubiquitin previously thought to be extremely rare, are also found within *Plasmodium*. First, a divergent ubiquitin sequence encoded by the UbL40 gene has only previously been found in *Giardia lamblia* (Catic and Ploegh 2005; but see supplementary text, Supplementary Material online). Here, we see that the UbL40-encoded ubiquitin has escaped from conservation on two independent occasions during the mammalian *Plasmodium* radiation, in the Vivax and Ovale lineages. Second, an extra codon inserted at the end of the ubiquitin coding repeats within the pUb gene was previously found in species of Cercozoa and Foraminifera (Archibald et al. 2003). This mutation was considered to be so rare, that this was taken as evidence that these two phyla likely shared a recent common ancestor, and interpreted as an indication that polyubiquitin processing in these organisms is unique among eukaryotes (Archibald et al. 2003). While the close relationship between Cercozoa and Foraminifera has been borne out in more recent analyses (Burki and Keeling 2013), we have found that a similar insertion has occurred quite recently in the common ancestor of *P. ovale-curtisi* and *P. ovale-wallikeri*. Seeing three occurrences of newly variant ubiquitin forms within the recent evolution of *Plasmodium* raises the question whether these malaria parasites are unusual in some way, or whether such events are in fact much more widespread across the eukaryote tree than previously realized.

Ubiquitin has many roles (Kwon and Ciechanover 2017; Dougherty et al. 2020), and it is unclear which, if any, of these roles the divergent forms of ubiquitin can fulfill. As noted above, the general extreme conservation of the ubiquitin sequence indicates that most amino acid replacements likely disrupt at least one aspect of the protein's function. Remarkably, Catic et al. (2007) found that the UbS27a-encoded “ubiquitin” from *G. lamblia* has very similar tertiary structure to that of human ubiquitin, despite very high sequence divergence. One of the functions of the ubiquitins encoded by the RP fusion genes is thought to be to chaperone the RP to the ribosome; Catic et al. (2007) suggested that the divergent *Giardia* protein may retain that particular function, but not others. Here, the UbL40-encoded ubiquitins in the Vivax and Ovale lineages were found to have accumulated amino acid changes at 15% (or more) of sites. However, as with the *Giardia* protein, the predicted tertiary structures of these divergent ubiquitins align very closely with the canonical ubiquitin structure. For example, the hydrophobic patch required for recognition of ubiquitin by the proteasome, formed by sites 8, 44, and 70 (Sloper-Mould et al. 2001), is retained with only a conservative substitution of valine by leucine in the Ovale lineage (fig. 3*a*). Ubiquitin is covalently attached to substrate proteins, thereby regulating their cellular fates. Proteins can be modified by a single ubiquitin or a polymeric chain, where linkages between ubiquitin monomers can be formed via any of seven lysine residues (Kwon and Ciechanover 2017). All seven of these residues (at sites 6, 11, 27, 29, 33, 48, and 63) are conserved in the UbL40-encoded ubiquitins from both Ovale species and most of the Vivax lineage (fig. 3*a*). The presence of all of these lysines involved in ubiquitin chain elongation, and the conservation of the overall structure, suggest that these divergent ubiquitins could retain some roles in post-translational modification.

The UbL40-encoded ubiquitin from the Ovale species also contains an additional lysine at position 54 (fig. 4). A lysine at this position, involved in forming functional linkages, has been described in two distinct cases of ubiquitin-related proteins. First, some insect viruses of the family Baculoviridae have a viral ubiquitin gene encoding a protein with about 76% identity to animal ubiquitin (Guarino 1990). Some of these viruses have this extra lysine at position 54, and it has been shown to be used as a linkage in polyubiquitin chains catalyzed (like eukaryotic ubiquitin) by the eukaryotic enzyme cascade of E1, E2, and E3 proteins (Negi et al. 2020). Second, eukaryotes encode a number of ubiquitin-like proteins, the most ubiquitin-like of which is NEDD8; human NEDD8 shares 58% identity with ubiquitin. In a comparison of NEDD8 sequences from 160 species, lysine occurred at position 54 in the great majority of species, although not in *Plasmodium* or other Apicomplexans (Bhattacharjee et al. 2020). In a mammalian cell line, this lysine was found to be involved in forming NEDD8-NEDD8 linkages (Vogl et al. 2020). Thus, it is possible that the additional lysine in the divergent Ovale ubiquitin could be involved in a new polyubiquitin linkage with novel functionality.

On the other hand, gene expression data suggest that the divergent UbL40-encoded ubiquitin in the Vivax lineage is not fully functional. In yeast it was found that under normal growth conditions, ubiquitin is mostly produced from the UbL40 and UbS27a genes, while expression of the pUb gene is induced under a variety of stress conditions (Finley et al. 1987); consistent with this, in yeast the UbL40 and UbS27a genes have synonymous codon usage similar to genes expressed at high levels, including other genes encoding RPs, while the pUb gene has much less biased codon usage (Sharp and Li 1987b). In *P. falciparum* it was also found that expression of the pUb gene increased following heat shock (Horrocks and Newbold 2000). Data taken from a recent study of the *P. falciparum* transcriptome over a period of 48 h (Chappell et al. 2020) indicate that during the first 24 h the UbL40 and UbS27a genes are expressed at much higher levels than the pUb gene, but that expression levels are more similar across the three genes over the second 24 h (supplementary fig. 3*a*, Supplementary Material online). In contrast, data taken from a recent study of the *P. vivax* transcriptome over a period of 48 h (Zhu et al. 2016) suggest that the pUb gene is consistently expressed at higher levels than either of the ubiquitin-RP fusion genes (supplementary fig. 3a, Supplementary Material online). This would be consistent with a need for higher expression of the pUb gene in *P. vivax* in order to provide enough fully functional ubiquitin, since both the UbL40 and the UbS27a genes encode divergent ubiquitins in that species (fig. 3*a*).

In comparisons of the polyubiquitin genes of *Plasmodium* species, numbers of nucleotide differences are lower between repeats within a species than between species, as previously described in other organisms (Sharp and Li 1987b; Tan et al. 1993; Keeling and Doolittle 1995; Zhou and Regan 1995; Nenoi et al. 1998). In retrospect, it is surprising that it has been suggested that ubiquitin genes have not undergone concerted evolution (Nei et al. 2000; Nei and Rooney 2005). Nei et al. (2000) suggested that the extent of synonymous substitution among ubiquitin repeats was so high as to preclude concerted evolution. However, even in the first description of the concerted evolution of ubiquitin genes, it was estimated that homogenization of sequences across the human polyubiquitin gene might have taken 40 million years (Sharp and Li 1987a), and nothing in the original description of concerted evolution (Zimmer et al. 1980) stipulates that the process must be rapid. Nei et al. (2000) concluded, instead, that the ubiquitin gene family has been subject to birth-and-death evolution. Unequal crossing-over between repeats is, in a sense, a birth-and-death process. However, unequal crossing-over has always been recognized as one of the processes leading to concerted evolution, along with gene conversion, and within the pUb loci it would be difficult to disentangle the relative contributions of these two processes. A tendency for the first repeats of the pUb genes to show less evidence of concerted evolution than the other repeats could reflect the presence of an intron within that repeat.

Concerted evolution resulting from exchange between the pUb and UbL40 loci is also apparent in *Plasmodium*. The Ser-Pro codon switch at residue 19 is consistently shared by the pUb and UbL40 genes across all five lineages of mammalian *Plasmodium* (fig. 3*a*). Given the phylogeny of these species (fig. 2), this switch, in both genes, appears to have occurred at least twice, and the most parsimonious explanation involves ectopic gene conversion between the two loci. The relatively low levels of synonymous nucleotide differences between the ubiquitin-encoding sequences of the UbL40 and pUb genes within the *Vinckeia* subgenus (fig. 6*c*) are particularly indicative of exchange between the loci within that lineage. Concerted evolution involving the UbL40 genes cannot be explained by birth-and-death processes because of the conserved fusion between the ubiquitin and RP coding sequences. Synteny, conserved across the *Plasmodium* species, between the ubiquitin coding and neighboring genes also seems quite inconsistent with birth-and-death processes.

In conclusion, this analysis of the ubiquitin genes of *Plasmodium* has confirmed the uniquely strong conservation of ubiquitin, but more interestingly has also revealed several occurrences of recent divergence in the encoded proteins, involving events that were previously thought to be very rare. This observation of both extreme conservation and divergence raises important questions about the functions of these proteins in this group of parasites. These *Plasmodium* genes also provide strong evidence of concerted evolution among the multiple copies of the ubiquitin coding sequence.

## Materials and Methods

Assembled *Plasmodium* genome sequences were obtained from GenBank using ACNUC (Perriere and Gouy 1996; the client retrieval program was obtained from https://doua.prabi.fr/databases/acnuc). Fifty-two complete genome sequences from 22 species of mammalian parasites, plus one from the bird parasite *P. relictum*, were analyzed. (This counts *P. malariae*-like, obtained from chimpanzees, as a separate species from *P. malariae* from humans; Rutledge et al. 2017; Plenderleith et al. 2022.) The locations of unannotated ubiquitin genes were found using BLAST searches (mainly tblastn). The accession numbers of all sequences, and locations of the ubiquitin genes, can be found in supplementary table S1, Supplementary Material online. For most analyses, only a single representative from each species was included.

Sequences were aligned and compared in ClustalW (Thompson et al. 1994). Dendrograms summarizing the similarity among repeats from different sources were constructed from observed levels of difference using the Neighbor-Joining method (Saitou and Nei 1987). Note that such a dendrogram is not a true phylogeny giving the evolutionary history of the repeats, because the recombination events implicated by the results are unlikely to have boundaries coinciding with the ends of the repeats. Phylogenetic relationships among species were estimated with a maximum likelihood analysis of 2,773 concatenated protein sequences (a total of 1,095,696 amino acid sites) encoded by one-to-one orthologues, using the Jones–Taylor–Thornton model with gamma-distributed rate variation and a class of invariant sites (JTT + G + I), using PhyML (Guindon et al. 2010). Figures were plotted using FigTree (http://tree.bio.ed.ac.uk/software/figtree/).

The crystal structure of human ubiquitin (1UBQ; Vijay-Kumar et al. 1987) was obtained from the RCSB Protein Data Bank (https://www.rcsb.org). The predicted structure of *P. falciparum* UbL40 (Q8ID50_PLAF7) was taken from the AlphaFold Protein Structure Database (www.alphafold.ebi.ac.uk; Jumper et al. 2021). For Ubl40 from *P. vivax* and *P. ovale-wallikeri*, ColabFold predictions were generated with the default MSA pipeline (UniRef + environmental), paired + unpaired mode, model type auto, and three cycles in the Google Colaboratory (Mirdita et al. 2022). The structures were imported into ChimeraX, and comparisons were performed using Matchmaker (Pettersen et al. 2021); the comparisons used the Needleman–Wunsch alignment algorithm with BLOSUM-62 similarity index, SS fraction 0.3, and iteration cutoff of 2.

## Supplementary Material

evad137_Supplementary_Data

## Data Availability

The sources of all data analyzed are listed in the Supplementary Material online.
