# Supplementary material for: Unusually Divergent Ubiquitin Genes and Proteins in Plasmodium Species
Source: Genome Biol Evol. 2023 Jul 22;15(8):evad137. doi: 10.1093/gbe/evad137 (PMC10457151; doi:10.1093/gbe/evad137)
Supplement: evad137_Supplementary_Data [file evad137_supplementary_data.zip › Supplement.pdf]

Unusually divergent ubiquitin genes and proteins in *Plasmodium* species

Thomas Dalhuisen, Lindsey J. Plenderleith, Ismail Ursani, Nisha Philip, Beatrice H. Hahn,

and Paul M. Sharp* (paul.sharp@ed.ac.uk)

Supplementary Material

Supplementary Figure 1
Alignment of the ubiquitin sequences encoded by the five repeats within the polyubiquitin (pUb) genes of *Plasmodium vivax* T01 and the two Ovale species.

Supplementary Figure 2
Alignment of the five ubiquitin coding repeats of the polyubiquitin (pUb) gene from *Plasmodium falciparum*.

Supplementary Figure 3
Expression of the three ubiquitin genes in *P. falciparum* and *P. vivax*.

Supplementary Tables are included in a separate file.

Supplementary Table 1
Details of the ubiquitin gene sequences analyzed.

Supplementary Table 2
The syntenic blocks surrounding the three ubiquitin genes in each genome.

Supplementary Table 3
Numbers of nucleotide differences between ubiquitin coding sequences.

Supplementary Table 4
Nucleotide composition of *Plasmodium* ubiquitin genes.

Supplementary Text
The ribosomal protein L40 genes of *Entamoeba histolytica*

Supplementary Figure 1

```
                         10        20        30        40        50        60        70
                          |         |         |         |         |         |         |
P. relictum pUb  MQIFVKTLTGKTITLDVESSDTIENVKAKIQDKEGIPPDQQRLIFAGKQLEDGRTLSDYNIQKESTLHLVLRLRGG
P. vivax pUb-1   ...........................S................................................
P. vivax pUb-2   ............................................................................
P. vivax pUb-3   ............................................................................
P. vivax pUb-4   ............................................................................
P. vivax pUb-5   ............................................................................F*
Ovale pUb-1      ........................................................L...................G
Ovale pUb-2      ............................................................................G
Ovale pUb-3      ............................................................................G
Ovale pUb-4      ............................................................................G
Ovale pUb-5      ............................................................................F*
```

Alignment of ubiquitin sequences encoded by the five repeats within the polyubiquitin (pUb) genes of *Plasmodium vivax* T01 and the two Ovale species (*Plasmodium ovale-curtisi* and *Plasmodium ovale-wallikeri*); only differences from the *Plasmodium relictum* sequence are shown. In the *P. vivax* gene the first repeat (only) encodes Ser at site 28. In the two Ovale species the first repeat (only) encodes Leu at site 57. As in all *Plasmodium* species, the fifth repeat is followed by a single sense codon (here encoding Phe) before the translation termination codon (*). Uniquely among *Plasmodium* species, in the two Ovale species the first four repeats have an additional codon, encoding Gly.

Supplementary Figure 2

```
consensus   ATGCAAATTTTTGTCAAAACATTAACAGGAAAAACAATAACTCTTGACGTCGAGCCATCT
pUb-1       ..........................T........T.....C..................
pUb-2       ............................................................
pUb-3       ............................................................
pUb-4       ............................................................
pUb-5       ............................................................

consensus   GACACCATTGAAAATGTTAAGGCAAAGATTCAAGATAAAGAAGGAATTCCACCTGACCAA
pUb-1       ........A..G................................................
pUb-2       ............................................................
pUb-3       ............................................................
pUb-4       ..........................A.................................
pUb-5       ............................................................

consensus   CAAAGATTAATATTTGCAGGAAAACAATTAGAAGATGGAAGAACTTTATCAGATTATAAC
pUb-1       ............................................................
pUb-2       ............................................................
pUb-3       ............................................................
pUb-4       .................T......................................C..T
pUb-5       ......................................T.....CC..........C..T

consensus   ATTCAAAAGGAATCTACATTACACTTAGTGTTAAGATTAAGAGGTGGT
pUb-1       ................................................
pUb-2       ................................................
pUb-3       ................................................
pUb-4       ..............C..TC..........A..................
pUb-5       ..............C..TC.............................TTTTAA
```

Alignment of the five ubiquitin coding repeats of the polyubiquitin (pUb) gene from *Plasmodium falciparum* 3D7 (accession number LN999947). The consensus sequence is shown at the top, and only differences from this are indicated for each repeat.

Supplementary Figure 3

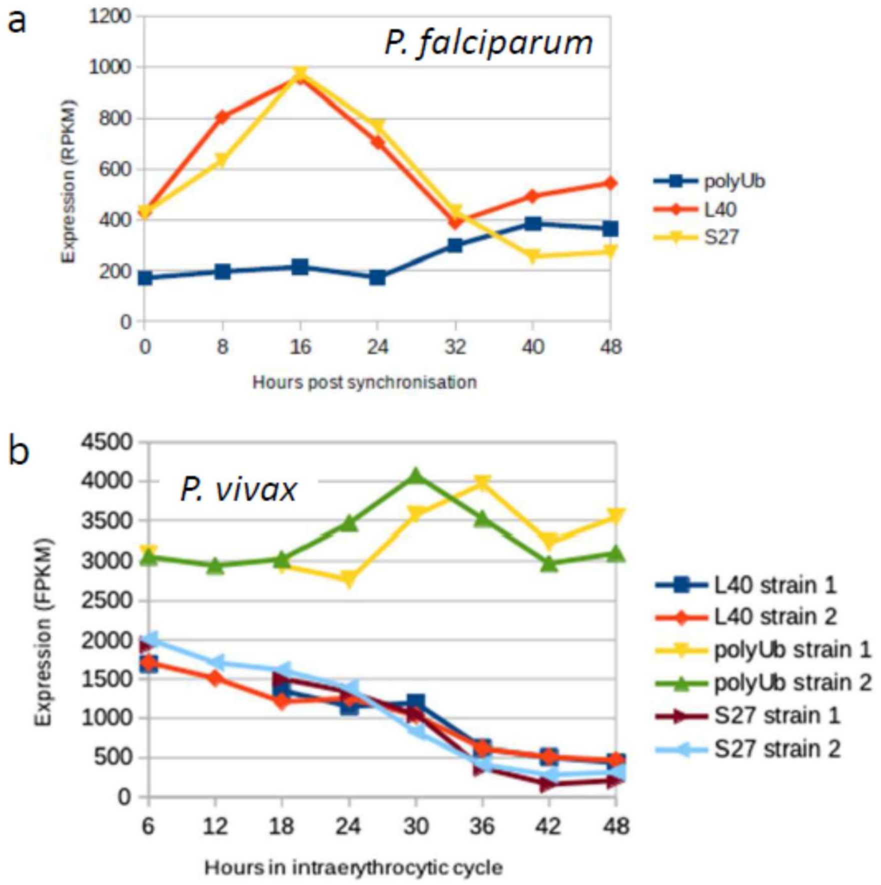

Expression of the three ubiquitin genes in (a) *P. falciparum*, and (b) *P. vivax*.

(a) Data for the expression of the *P. falciparum* polyubiquitin (blue), ubiquitin-RPL40 (red) and ubiquitin-RPS27a (yellow) fusion genes at different time points, taken from Chappell et al. (2020). The data were generated by synchronising cell cultures of *P. falciparum* strain 3D7 and obtaining directional, amplification-free RNA-Seq libraries from RNA samples taken at eight-hourly intervals. Values for the expression of each gene at each time point in reads per kilobase per million reads mapped (RPKM) were obtained from Chappell et al. Additional file 4 (Table S3).

(b) Data for the expression of the *P. vivax* polyubiquitin (yellow and green), ubiquitin-RPL40 (dark blue and red) and ubiquitin-RPS27a (magenta and light blue) fusion genes at different time points, taken from Zhu et al. (2016). RNA-Seq data were generated from two highly synchronous clinical isolates from Northwestern Thailand, maintained as *ex vivo* cultures for 48 h with RNA samples taken at six-hourly intervals. Values for the expression of each gene at each time point in fragments per kilobase of coding exon per million fragments mapped (FPKM) were obtained from PlasmoDB (v47).

Supplementary Text

The ribosomal protein L40 genes of *Entamoeba histolytica*

Catic and Ploegh (2005) noted 13 species in which the "ubiquitin" encoded by the ubiquitin-ribosomal protein S27a fusion gene (UbS27a) was highly divergent. These included three species of Apicomplexa (*Plasmodium falciparum*, *Toxoplasma gondii*, and *Cryptosporidium hominis*), as well as 5 other protozoans (*Entamoeba histolytica*, *Giardia lamblia*, *Leishmania major*, *Trypanosoma cruzi, Trichomonas vaginalis*), a green alga (*Chlamydomonas reinhardtii*), a red alga (*Cyanidioschyzon merolae*), a fungus (*Cryptococcus neoformans*) and two animals (*Ancylostoma ceylanicum* and *Caenorhabditis elegans*, both nematodes). In five other species (*Homo sapiens*, the nematode *Ascaris suum*, the yeast *Saccharomyces cerevisiae*, a plant *Zea mays*, and a slime mould *Dictyostelium discoideum)* the ubiquitin encoded by the UbS27a gene was conserved.

Catic and Ploegh (2005) also indicated that the "ubiquitin" encoded by the ubiquitin-ribosomal protein L40 fusion gene (UbL40) was divergent in two of these 18 species, *Entamoeba histolytica* and *Giardia lamblia*. However, only the case of *Giardia lamblia* seems accurate.

The *Entamoeba histolytica* genome (Loftus et al. 2005) contains four sequences of 57 codons, on four different chromosomes, encoding 56 residue proteins with up to 80% identity to ribosomal protein L40 sequences from a variety of other phyla. These are:

Chromosome 1: AP023109.1 sites (c)1497776-1497946.

Chromosome 6: AP023114.1 sites 909250-909420.

Chromosome 12: AP023120.1 sites 296176-296346.

Chromosome 13: AP023121.1 sites 692372-692542.

These four putative genes show 8-19 nucleotide differences, all synonymous. Residues 3-53 show 80% identity (41/51) to the ribosomal protein L40 sequences encoded by ubiquitin-ribosomal protein L40 fusion genes from (for example) *Neohortaea acidophila* (an ascomycete fungus) and *Naegleria fowleri* (an amoeboflagellate excavate protist). Nucleotide sequences upstream of these RPL40 coding sequences are not conserved among the four copies, and none of the four has sequences detectable as encoding a sequence related to ubiquitin. While the first four residues of (as an example) human RPL40 are Ile-Ile-Glu-Pro, the first five residues encoded by these Entamoeba genes are Met-Gly-Ile-Glu-Pro; that is, an additional Met codon appears at the 5' end of the sequences, which can provide a translational start site for production of the ribosomal protein in the absence of read through from an upstream ubiquitin coding sequence.

The sequence identified by Catic and Ploegh (2005) as a putative divergent ubiquitin encoded by a ubiquitin-ribosomal protein L40 fusion gene is 54 amino acids long. The first 51 residues are encoded by nucleotides 295862-296014 on chromosome 12 (AP023120.1).

Nucleotides 296015-296016 are GT, which could be the start of an intron. Residues 52-54 of the sequences presented by Catic and Ploegh (2005) are Thr-Thr-Thr. The 11 nucleotides 5' of the putative ATG start codon of the RPL40 gene on chromosome 12 are AG (which could be the end of an intron) followed by three Thr codons. Thus, it appears that Catic and Ploegh (2005) envisaged an intron from

296017 to 296166, near the end of the putative ubiquitin-coding region, as found in ubiquitin-ribosomal protein L40 fusion genes. However, even after the insertion of five gaps in an alignment, there are only 12 potential identities between this putative ubiquitin sequence and that encoded by the *E. histolytica* “polyubiquitin” gene, and there are no related sequences upstream of the other three copies of the RPL40 gene. Thus, it seems likely that there is no ubiquitin-ribosomal protein L40 fusion gene in this species.

References

Catic A, Ploegh HL. 2005. Ubiquitin – conserved protein or selfish gene? Trends Biochem Sci 30:600-604.

Chappell L, et al. 2020. Refining the transcriptome of the human malaria parasite *Plasmodium falciparum* using amplification-free RNA-seq. BMC Genomics 21:395.

Loftus B, et al. 2005. The genome of the protest parasite *Entamoeba histolytica*. Nature 433:865-868.

Zhu L, et al. 2016. New insights into the *Plasmodium vivax* transcriptome using RNA-Seq. Scientific Reports 6:20498.

Supplementary Tables are included in a separate file.

Supplementary Table 1

Details of the ubiquitin gene sequences analyzed. The table gives the chromosome number, the GenBank/NCBI accession number of the chromosome sequence, and the position of the nucleotide at the start and the end of the gene. For the polyubiquitin gene (pUb) the number of ubiquitin coding repeats, and the amino acid encoded by the extra codon at the 3' end, are also indicated. These details are given for 53 genome sequences; the 23 genomes included in the analyses are indicated.

Supplementary Table 2

The syntenic blocks surrounding the three ubiquitin genes in each genome. The table gives the identity of the orthologues at each end of the block, the identity of the ubiquitin genes, and the number of genes to either side of the ubiquitin genes, for representative species from each of the five major lineages (*P. falciparum*, *P. vivax*, *P.malariae*, *P. ovale-wallikeri* and *P. berghei*) as well as the outgroup species (*P. relictum*).

Supplementary Table 3

Numbers of nucleotide differences between ubiquitin coding sequences. Values given are for (i) the average number of differences between the various repeats within the polyubiquitin gene of each species (ii) the average number of differences between the ubiquitin coding region of the UbL40 gene and the various repeats within the polyubiquitin gene of the same species, (iii) the average number of differences between the various repeats within the polyubiquitin gene of each pair of species, and (iv) the number of nucleotide differences between the ubiquitin coding region of the UbL40 genes of each pair of species.

Supplementary Table 4

Nucleotide composition of *Plasmodium* ubiquitin genes. Values given are for G+C content at synonymously variable third positions of codons (GC3s).
